# Preparation of Nanoemulsions of Vitamin A and C by Microfluidization: Efficacy on the Expression Pattern of Milk-Specific Proteins in MAC-T Cells

**DOI:** 10.3390/molecules24142566

**Published:** 2019-07-15

**Authors:** Tae-Il Kim, Tae-Gyun Kim, Dong-Hyun Lim, Sang-Bum Kim, Seong-Min Park, Tai-Young Hur, Kwang-Seok Ki, Eung-Gi Kwon, Mayakrishnan Vijayakumar, Young-Jun Kim

**Affiliations:** 1 Dairy Science Division, National Institute of Animal Science, Rural Development Administration, Chungcheongnam-do, Cheonan 31000, Korea; 2 Department of Food and Biotechnology, Korea University, 2511 Sejong-ro, Sejong 30019, Korea; 3 Hanwoo Research Institute, National Institute of Animal Science, Rural Development Administration, Pyeongchang-gun, Gangwon-do 25340, Korea

**Keywords:** nanoemulsion, microfluidization, vitamins, casein

## Abstract

In this study, we prepared stabilized vitamin A and C nanoemulsions, and investigated their efficacy on milk-specific proteins in bovine mammary epithelial cells (MAC-T). Emulsions of vitamin A (vit-A) and C (vit-C) were prepared using Lipoid S 75 and microfluidization. The particle size and polydispersity index (PDI) of nanoemulsified vit-A and vit-C were studied. The cytotoxic effect of nanoemulsion-free and nanoemulsified vit-A and vit-C was determined by an MTT assay. In addition, the efficacy of nanoemulsified vit-A and vit-C on the in vitro expression pattern of milk-specific proteins in MAC-T cells was investigated by quantitative RT-PCR. The results showed that the efficacies of stabilized nanoemulsions of vit-A and vit-C were 100% and 92.7%, respectively. The particle sizes were around 475.7 and 225.4 nm, and the zeta potentials were around −33.5 and −21.3 mV, respectively. The expression changes of α_s2_-, β- and κ-casein were higher in the presence of a stabilized nanoemulsion of vit-A, compared with nanoemulsion-free vit-A. Furthermore, the expression changes of α_s2_- and β-casein were lower and that of κ-casein was higher in the presence of a stabilized nanoemulsion of vit-C, compared with nanoemulsion-free vit-C. Thus, our findings demonstrate the efficacy of nanoemulsified vit-A and vit-C in changing the expression of milk-specific proteins in MAC-T cells.

## 1. Introduction

Nanotechnology is one of the most promising and rapidly growing fields, with great potential in the field of science and technology [1,2]. Nanotechnology plays a major role in the control of materials ranging in size from 1 to 100 nm. Prepared or assembled nanosized (10^−9^ m) materials can provide commercial, technological, and scientific opportunities for industry [1]. They have great potential for applications in the fields of drug delivery, sensor systems, textile industry, cosmetics, and food industry. In particular, nanosized materials can modify the texture, taste, sensory attributes, color, strength, and stability of foods during shelf life. Furthermore, they can improve water solubility, thermal stability, and the oral bioavailability of functional compounds [1,3,4,5]. Several bioactive molecules (in the form of capsules or tablets) are available in the market and used for providing health benefits, such as prevention or treatment of diseases [6]. However, it has become evident that solutions of these molecules may not maintain the health benefits of the functional compounds, particularly in the case of nonpolar compounds [7,8]. Enhancement of the bioavailability of bioactive molecules is essential for improving their absorption in the gastrointestinal tract. The development of nanotechnology can offer possible solutions for improving the water solubility and bioavailability of (lipophilic) functional compounds [6].

Nanoemulsions can be defined as thermodynamically unstable nanodroplets made by two immiscible liquids (oil in water or water in oil), with the average droplet size ranging from 1 to 500 nm. The nanosize has useful benefits such as high surface area, stability, transparent appearance, and tunable rheology. Nanoemulsions have exciting applications in diverse areas, including foods, pharmaceuticals, nutraceuticals, drug delivery, flavors, antioxidants, cosmetics, firefighting, and material synthesis [9,10,11]. Usually, the lipid phase is dispersed in an aqueous phase of the emulsion, and each oil droplet is being surrounded by a thin interfacial layer of emulsifier molecules [10,12,13]. These nanosized particles are stable to gravitational separation due to Brownian motion effects, and they have excellent stability against droplet aggregation due to the range of repulsive forces between the droplets and particles [13]. Several methods such as emulsification–evaporation, emulsification–diffusion, solvent displacement, and precipitation have been extensively used to prepare lipophilic functional substances with particles in the nanosize range by using high- or low-energy methods [12]. The high-energy methods use high mechanical energy, which produces more controlled sizes and compositions of the materials. However, some chemicals are easily degraded during preparation. On the other hand, a low-energy method requires low energy for the production of the nanoemulsion, and it depends mainly on the internal factors of the surfactants and the oily phase [14]. 

Vitamins are regarded as essential for reproduction, milk production, and milk component yield because of their major role in cellular metabolism and growth. In addition, they have a unique mechanism of action, and so they are needed for dairy cows. Many of these vitamins have antioxidant activities, which are beneficial to animal health and milk production. When the level of vitamins decreases around calving, more supplementation is recommended in animals. However, their need varies, for example depending on the quality of the food in the diet. In the past few years, many researchers have focused on understanding the impact of vitamins on milk production and milk component yield in dairy cows [15,16,17]. Vit-A plays a vital role in the maintenance of epithelial tissue, in mucosal surface integrity and stability [18], and in the control of pathogen entry into the mammary gland. A previous study also confirmed that the supplementation of vit-A has an impact on the udder when the plasma level is decreased below 0.4 mg/L [19]. Vit-C is a water-soluble antioxidant that is essential for humans, guinea pigs, ruminants, swine, horses, dogs, and cats for the production of ascorbic acid from glucose in the liver, and for birds for the production of ascorbic acid in the liver or the kidney. The dietary need of vit-C is not confirmed in animals that can produce ascorbic acid. However, the prevalence of scurvy in dairy cattle is a typical sign of lack of vit-C [20]. Vit-C plays a significant role in neutrophil production in response to free radical attack [21], in cell protection against viral attack by stimulating interferon production [22], and in preventing the formation of nitrosamine [23]. Also, vit-C stimulates the in vitro differentiation of myoblasts [24], osteoblasts [25], and adipocytes [26], which are all mesenchyme-derived cells. Mammary epithelial cells (MAC-T) play an important role in milk production and milk component yield, which are evaluated by the metabolic activity and number of these cells in the mammary gland [27]. To the best of our knowledge, there is no study on the impact of nanoemulsion-based delivery of vitamins on milk-related proteins in MAC-T cells. Therefore, the aim of the present research was to investigate the effect of nanoemulsified vit-A and vit-C on the in vitro expression changes of milk-related proteins in MAC-T cells.

## 2. Results

### 2.1. Effect of Microfluidization Condition, Efficiency, Emulsion Size, and Zeta Potential of Vit-A and -C Nanoemulsion

**Figure 1:**
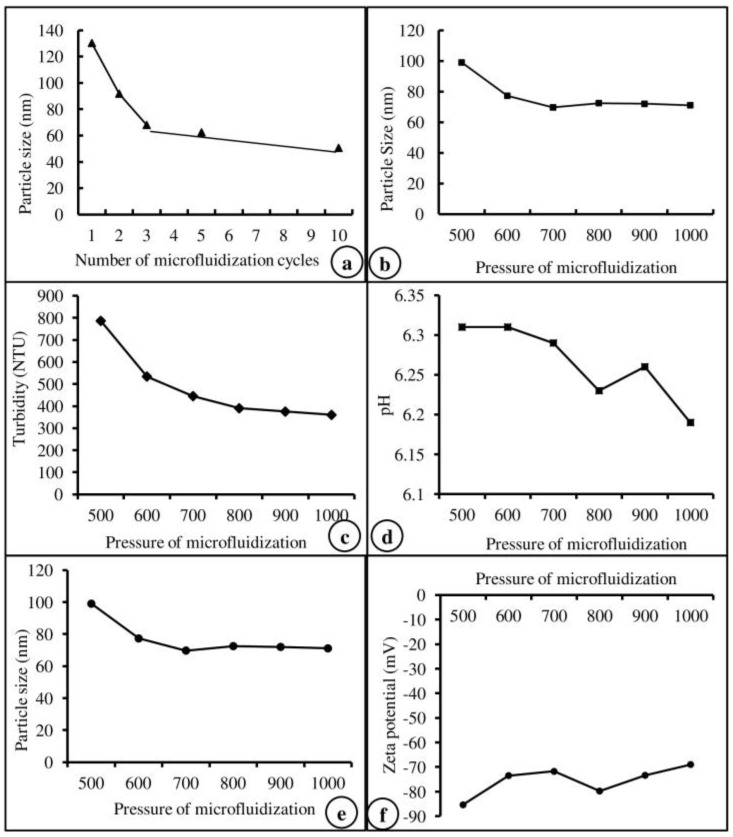
Physiological characteristics of microfluidization cycles (**a**), pressure (**b**), turbidity (**c**), pH (**d**), particle size (**e**), and zeta potential (**f**) of the core material (Lipoid S75).

Based on the results of our previous study on efficiency and particle size [28], the number of microfluidization cycles (Figure 1a), the pressure of microfluidization (Figure 1b), and the core material of Lipoid S 75 were selected and used in this study. The physiological characteristics of the core material, such as turbidity, pH, emulsion size, and zeta potential, are represented in Figure 1c–f. Based on the physiological characteristics, Lipoid S 75 was selected and used to prepare the nanoemulsion of vitamins. The encapsulation efficiency of the core material used for vitamin nanoemulsion was calculated from the total amount of vit-A and -C in the nanoemulsion and the amount of vitamin extracted from the surface of the nanoemulsion. Vit-A was entrapped in unsaturated soybean lecithin (Lipoid S 75) with an encapsulation efficiency of 100%, whereas vit-C was entrapped in unsaturated soybean lecithin (Lipoid S 75) with an encapsulation efficiency of 92.7%. 

**Figure 2:**
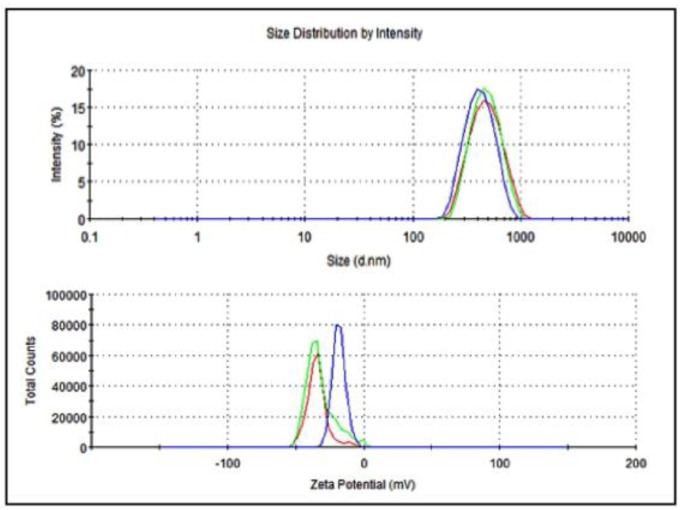
Particle size and zeta potential distribution of nanoemulsified vit-A by intensity using dynamic light scattering correlation spectroscopy. The average particle size of nanoemulsified vit-A was around 475.7 nm, and the zeta potential of nanoemulsified vit-A was around −33.5 mV. The polydispersity index (PDI) value of the nanoemulsified vit-A was 1.70.

**Figure 3:**
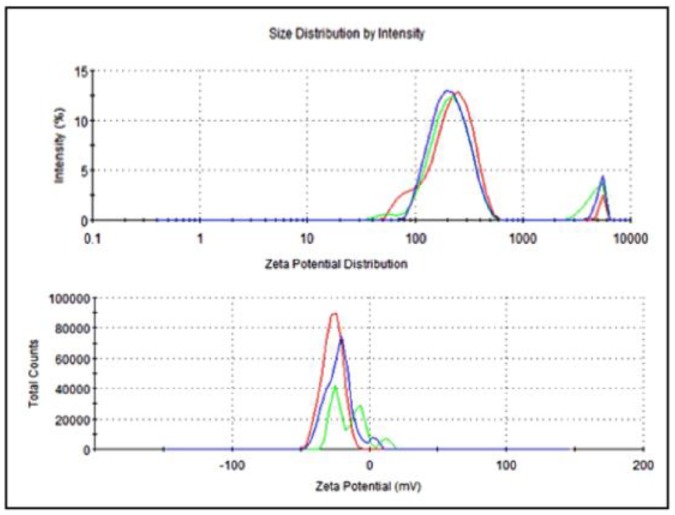
Particle size and zeta potential distribution of nanoemulsified vit-C by intensity using dynamic light scattering correlation spectroscopy. The average particle size of nanoemulsified vit-C was around 225.4 nm, and the zeta potential of nanoemulsified vit-C was around −21.3 mV. The PDI value of the nanoemulsified vitamin C was 0.81.

The average particle size of nanoemulsified vit-A was around 475.7 nm, and the zeta potential of the vit-A nanoemulsion was around −33.5 mV (Figure 2). Similarly, the average particle size of the nanoemulsified vit-C was around 225.4 nm, and the zeta potential of the vit-C nanoemulsion was around −21.3 mV (Figure 3). These low values may be due to the adsorption of light by vitamins.

### 2.2. Effect of Nanoemulsified Vitamins on MAC-T Cell Growth and Viability

**Figure 4:**
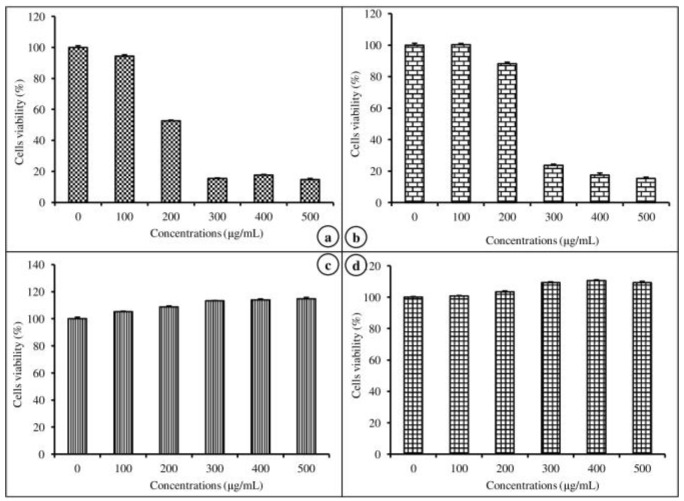
Viability of mammary epithelial cells (MAC-T) after 48 h of exposure to different concentrations of nanoemulsified vitamins (100, 200, 300, 400, and 500 μg/mL). Cell viability is expressed as the percentage of emulsion-free vit-A (**a**), nanoemulsified vit-A (**b**), emulsion free vit-C (**c**), and nanoemulsified vit-C (**d**), as the average (±SEM of the average) of three independent experiments.

The cell viability efficiency of nanoemulsion-free and nanoemulsified vitamins on MAC-T was determined by an MTT assay at various concentrations (100–500 μg/mL) for 24 h. The cell viability potential of the emulsion-free and nanoemulsified vitamins was expressed as a percentage, calculated from the ratio of the number of living cells treated with the nanoemulsified vitamins to the number of living cells treated with the emulsion-free vitamins. As shown in Figure 4a,b, there was no significant difference between the control and the 100 μg/mL concentration of emulsion-free and nanoemulsified vit-A, but at a concentration of 200–500 μg/mL of nanoemulsified vit-A, the cell viability significantly decreased (*p* < 0.05). Similarly, for all tested concentrations (100–500 μg/mL) of emulsion-free and nanoemulsified vit-C, the cell viability was shown to be above 100% of the control (Figure 4c,d). Our study results confirmed the impact of emulsion-free and nanoemulsified vitamins on cell viability. From the above findings on cell viability, the 100 mg/mL concentration was selected for further study on how nanoemulsified vitamins affect the expression changes of casein mRNA in MAC-T cells.

### 2.3. Effects of Nanoemulsified Vitamins on the In Vitro Expression Changes of Casein mRNA in MAC-T Cells

**Figure 5:**
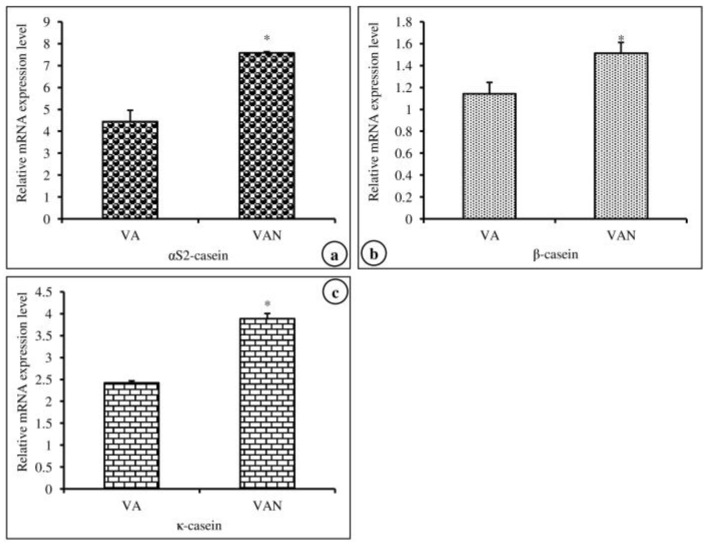
Effect of nanoemulsion-free vit-A (VA) and nanoemulsified vit-A (VAN) on casein gene expression in MAC-T cells as evaluated by quantitative RT-PCR. The nanoemulsified vit-A had stimulatory effects on the α_s2_- (**a**), β- (**b**), and κ-casein (**c**) expression when compared with the non- nanoemulsified vitamins, as the average (±SEM of the average) of three independent experiments.

**Figure 6:**
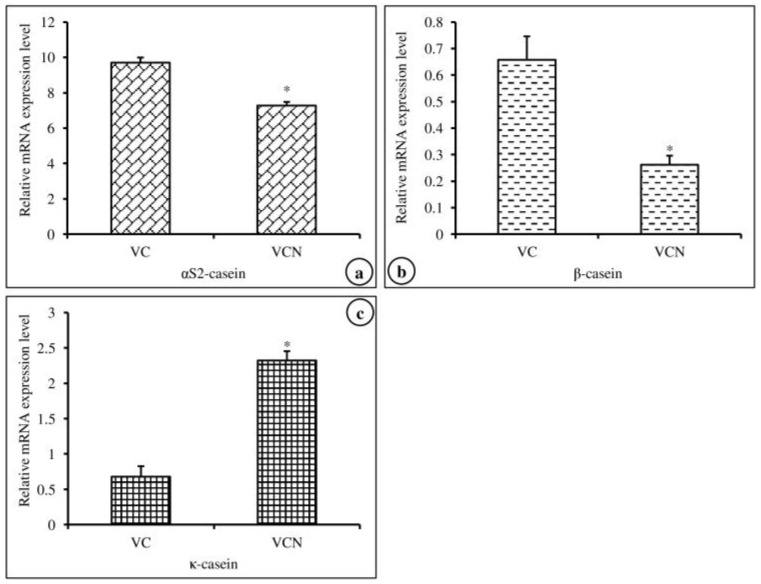
Effect of nanoemulsion-free vit-C (VC) and nanoemulsified vit-C (VCN) on casein gene expression in MAC-T cells as evaluated by quantitative RT-PCR. The nanoemulsified vit-C had inhibitory effects on α_s2_- (**a**), β- (**b**), and κ-casein (**c**) expression and stimulatory effects on κ-casein expression when compared with the non-nanoemulsified vitamins, as the average (±SEM of the average) of three independent experiments.

In this experiment, we investigated the effects of nanoemulsified vit-A and vit-C on the in vitro expression changes of casein mRNA in MAC-T cells. The results of the relative mRNA expression quantities of the α-, β-, and κ-casein genes are displayed in Figure 5a–c and revealed that the synthesis of α_s2_-, β-, and κ-casein in nanoemulsified vit-A-treated cells was significantly higher than that in nanoemulsion-free vit-A-treated cells. In contrast, the results of the current study showed that the expression level of α- and β-casein mRNA was decreased and that of κ-casein mRNA was increased in nanoemulsified vit-C-treated cells when compared to nanoemulsion-free vit-C-treated cells (Figure 6a–c).

## 3. Discussion

The aim of this investigation was to establish the bioavailability of nanoemulsified vitamin A and C by nanoemulsion. This was achieved by providing the particle size distribution and zeta potential of the nanoemulsion as measured by static and dynamic light scattering techniques [29]. As shown by the results of the current study, these reduced sizes have good stability. In addition, our results confirmed that high-pressure homogenization three times with 1000 psi was the optimal condition for preparing nanoemulsified vitamins. Generally, the zeta potential characterizes the surface charge of the nanoemulsified particle. High absolute values of the particle lead to repulsive forces between the particles, which improve the physical stability of the multiphase system. The zeta potential of the nanoemulsified vitamins ranged from −20 to −48.1 mV, indicating good stability; values above 60 mV indicate excellent long-term stability of the nanoemulsified particles [30]. The polydispersity of the particles is caused by the core condition of dynamic light scattering, due to a high concentration in the cuvette. This leads to multiple scattering with decreased path lengths, a result that involves Brownian motion effects. These circumstances can enable the achievement of proper particle sizes. A possible reason may be that Lipoid S 75 can attract water, so more water is added; therefore, the dynamic diameter increases slightly. As the sample becomes more diluted, more Lipoid S 75 may move out of the particle, causing the particle size to decrease. Fortunately, this is not the case, as this can lead to substantial problems when using nanoemulsions in therapeutic applications [31]. 

A calorimetric MTT assay was carried out 24 h after the preparation of nanoemulsion-free or nanoemulsified vitamin A and C to determine the possible cytotoxic activity of these nanoemulsions. Our results showed that cell proliferation increased with increasing concentration of cells treated with nanoemulsified vitamins when compared to cells containing nanoemulsion-free vitamins. However, the cells were slightly more disturbed by the treatment with nanoemulsified vitamins than by the treatment with nanoemulsion-free vitamins. These differences may be due to the effects of vitamins on the cell cycle or apoptosis or both; additional cell cycle and apoptosis analysis may address this issue. 

Casein and whey are the major milk-specific proteins in milk. These two proteins are excellent sources of all amino acids, minerals, antibodies, inorganic calcium, and phosphate, but they differ in one aspect in that whey is a fast-assimilating protein and casein is a slow-assimilating protein. Casein is hydrophobic and can form casein micelles. These micelles contain most, but not all, of the casein proteins, and casein proteins can also transport calcium, inorganic phosphate, citrate, and magnesium and act as a nutritional source for amino acids such as proline and glutamic acid [32]. During lactation, free amino acids express the casein gene predominantly in the ribosomes of the mammary gland [33]. The Golgi apparatus plays a major role in the maturation of casein micelles and delivers the proteins into vesicles for secretion. Furthermore, casein has three subtypes: α-, β-, and κ-casein [32]. 

Casein could be a good indicator for epithelial cell differentiation in the mammary gland. During bovine mammary epithelial cell differentiation by hormonal stimulation of transcription, the expression of casein mRNAs significantly increased [34]. Choi et al. [33] confirmed that mammary cells were capable of hormone-induced milk-specific protein expression. A mixture of hydrocortisone, prolactin, and insulin enhanced the level of milk-specific protein secretion. Moreover, Hobbs et al. [35] reported that the addition of prolactin can enhance the secretion of milk proteins due to the observed increased level of β-casein mRNA. These authors also proved that prolactin plays a major role in the transcription or turnover rate of casein mRNA expression [35]. Insulin may not be essential for the expression of casein in MAC-T cells because it has the lowest effect on casein expression [36]. Casein is an important milk-specific protein in mature milk. Moreover, a previous study showed that casein production and secretion indicate complete mammary epithelial differentiation of lactation [37]. The mRNA expression of the α-casein and β-casein proteins is used as an indicator of the differentiation of MAC-T cells [38]. The existence of these proteins can be due to the secretory capability of MAC-T cells. Taken together, the results of our study provide a nanoencapsulation basis for the use of vitamins in regulating milk-specific proteins in MAC-T cells. To our knowledge, this is the first report on the impact of nanoencapsulated vit-A and vit-C on the expression pattern of milk-specific proteins in MAC-T cells. Further work is needed to better understand the molecular mechanism by which vit-A and vit-C supplementation influence the regulation of milk-specific proteins in MAC-T cells.

## 4. Materials and Methods

### 4.1. Cell Culture and Chemicals

Bovine mammary epithelial (MAC-T) cells (ATCC: CRL-10274) were purchased from the American Type Culture Collection (Rockville, MD, USA). Dulbecco’s modified Eagle medium (DMEM), fetal bovine serum (FBS), and antibiotics were obtained from Gibco-BRL (Gaithersburg, MD, USA). The mRNA extraction (iNtRON Biotechnology, Inc., Gyeonggi-do, Korea) and RT-PCR kits were procured from Invitrogen (Carlsbad, CA, USA). 3-(4,5-Dimethylthiazol-2-yl)-2,5- diphenyltetrazolium bromide (MTT) was purchased from Amresco (Solon, OH, USA). Insulin was purchased from Sigma Aldrich (St. Louis, MO, USA). Lipoid S 75, Lipoid S 100, Lecinol S 10, hydrogenated soy phosphatidylcholine (HSPC 50), Lipoid S 75-3, and Lipoid S 100-3 were obtained from Pharmachem (Seoul, Korea). All other chemicals and solvents used were of analytical grade. 

### 4.2. Preparation of Vit-A and Vit-C Nanoemulsion

**Figure 7:**
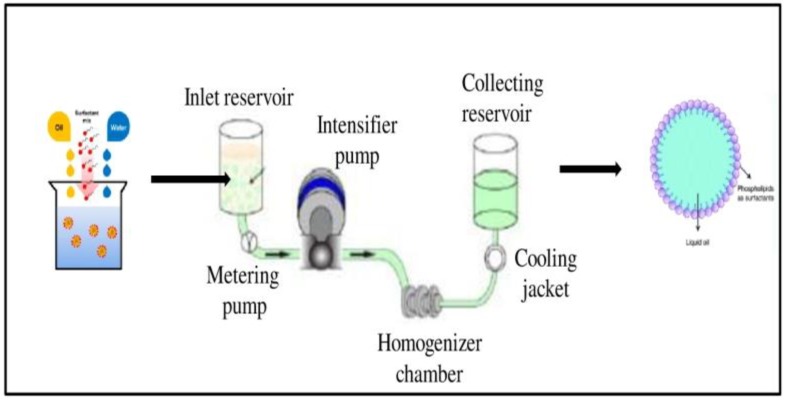
Preparation of nanoemulsion of vitamin A and C using Lipoid S75 and microfluidization.

Nanoemulsions of vit-A and vit-C were prepared using lecithin (Lipoid S 75) and microfluidization with the high-pressure homogenization (HPH) method. Briefly, 10 wt. % of vit-A and vit-C (separately) was mixed with 10 wt. % of ethanol, and then the mixture was added with 5 wt. % of soybean lecithin, 75 wt. % of water. The core emulsion was mixed by a high-shear mixing homogenizer at 24,000 rpm for 4 min to get a primary emulsion, and the final emulsion was achieved by high-speed homogenization using an Ultra Turrax T25 blender (IKA Labortechnik, Germany). Then, the cooled pre-emulsion was passed through a high-pressure homogenizer (MN400BF, Micronox) at 1000 psi three times, resulting in nanoemulsified vit-A and vit-C. The temperature for the complete homogenization process was maintained below 40 °C. The collected nanoemulsified vit-A and vit-C were stored at −80 °C until further use (Figure 7).

### 4.3. Determination of Droplet Size (Diameter), Polydispersity Index (PDI), and Zeta Potential (ζ-Potential)

The measurement of droplet size and the polydispersity (zeta potential) index of the nanoemulsified vit-A and vit-C was done by dynamic light scattering correlation spectroscopy using a high-performance particle sizer (HPPS) (Malvern Instruments, Malvern, UK). The droplet size distribution was described in terms of mean droplet size (Z-diameter) and width of the distribution (polydispersity index, PDI) at a scattering angle of 190° at 25 °C. Briefly, by measuring the backscattered light of 100 μL samples mixed with 1 mL of distilled water, put into a polystyrene latex cell and measured at a scattering angle of 190°, we obtained a dispersant refractive index of 1.33 and a material refractive index of 1.59 at 25 °C. 

### 4.4. Analysis of Cell Growth and Viability

Determining the cell viability potential of nanoemulsified vitamin A and C on MAC-T cells was done by an MTT assay. Briefly, cells were seeded in a 96-well plate at a density of 1 × 10^4^ cells/well, and cell plates were incubated in a humidified atmosphere of 5% CO_2_ at 37 °C. After a 24 h interval, cells were treated with different concentrations (100–500 μg/mL) of nanoemulsified vit-A and vit-C, respectively. Following 48 h of incubation at 37 °C with 5% CO_2_, cells were treated with 10 μL of MTT solution and incubated for 3 h. The optical density of each well was measured at a wavelength of 450 nm using a Spectra count ELISA (enzyme-linked immunosorbent assay) plate reader (Packard Instrument Co., Downers Grove, IL, USA). 

### 4.5. Investigating the Efficacy of Vit-A and Vit-C Nanoemulsion on the In Vitro Expression of Milk-Specific Proteins

Mammary epithelial cells were seeded in a 12-well culture plate at a density of 2 × 10^4^ cells/well in DMEM (supplemented with 10% of FBS, 100 μg/mL of penicillin and streptomycin, 5 μg/mL of insulin, 50 μg/mL of gentamicin, and 1 μg/mL of hydrocortisone) medium added to the culture plates, and culture plates were kept in a humidified atmosphere, including 5% CO_2_ at 37 °C. After 100% confluency, cells were treated with nanoencapsulated vitamins at a concentration of 100 mg/mL for 24 h. The experiment was conducted independently three times. The cellular RNA was isolated from MAC-T cells using the RNeasy lipid tissue kit (Qiagen, Valencia, CA, USA). The isolated RNA was quantified using a UVS-99 microvolume UV/Vis spectrometer (ACT gene, Piscataway, NJ, USA). The cDNA synthesis was carried out using 500 ng of cellular RNA with oligo (dT) primers and reverse transcriptase provided by the Superscript III first-strand synthesis system for RT-PCR (Invitrogen). The level of mRNA expression of milk proteins (casein) was assessed by SYBR Green-based real-time PCR on an ABI 7500 PCR system (Applied Biosystem, Foster City, CA, USA). The target gene expression levels were normalized with housekeeping genes. Primers used for qPCR are listed in Table 1. 

### 4.6. Statistical Analysis

All the samples were prepared in triplicate. Data were statistically analyzed, and a comparison of the means was performed using Excel and a one-way ANOVA with the statistical package SPSS-16.0 (SPSS, Inc., Chicago, IL, USA). The results were expressed as mean ± SE of the mean, and statistical significance was set at *p* < 0.05. 

## 5. Conclusions

To the best of our knowledge, this is the first report on the efficacy of nanoemulsified vit-A and vit-C on the expression pattern of milk-specific proteins in MAC-T cells. The results of the present study showed that the core material used for the preparation of nanoemulsified vitamins improved the efficiency, size, zeta potential, and PDI of the emulsified vitamins. Furthermore, the supplementation of nanoemulsified vitamins to MAC-T cells demonstrated that nanoemulsified vitamins are potentially more effective at influencing milk-specific protein synthesis than nanoemulsion-free vitamins. Further research is needed to better understand the molecular mechanism by which vit-A and vit-C supplementation affects the regulation of milk-specific proteins in in vitro and in vivo models.

## Figures and Tables

**Table 1:** Oligonucleotide primer sets for real-time PCR.

Gene	Forward Primer (5′-3′)	Reverse Primer (3′-5′)	Source
αs2 casein	AGCTCTCCACCAGTGAGGAA	GCAAGGCGAATTTCTGGTAA	NM_174528.2
β casein	GTGAGGAACAGCAGCAGCAAACA	TTTTGTGGGAGGCTGTTAGG	NM_181008
κ casein	CCAGGAGCAAAACCAAGAAC	TGCAACTGGTTTCTGTTGGT	NM_174294
GAPDH	GGGTCATCATCTCTGCACCT	GGTCATAAGTCCCTCCACGA	XM_001252479
